# Virus-Mimicking Polymer Nanocomplexes Co-Assembling HCV E1E2 and Core Proteins with TLR 7/8 Agonist—Synthesis, Characterization, and In Vivo Activity

**DOI:** 10.3390/jfb16010034

**Published:** 2025-01-19

**Authors:** Thomas R. Fuerst, Alexander Marin, Sarah Jeong, Liudmila Kulakova, Raman Hlushko, Katrina Gorga, Eric A. Toth, Nevil J. Singh, Alexander K. Andrianov

**Affiliations:** 1 Institute for Bioscience and Biotechnology Research, University of Maryland Rockville, Rockville, MD 20850, USA; 2 Department of Cell Biology and Molecular Genetics, University of Maryland, College Park, MD 20742, USA; 3 Department of Microbiology & Immunology, University of Maryland School of Medicine, Baltimore, MD 21201, USA

**Keywords:** polyphosphazenes, polymer nanocomplexes, vaccine delivery, hepatitis C virus, immunoadjuvants, supramolecular assembly, resiquimod, immunoadjuvants, PEGylated polymer

## Abstract

Hepatitis C virus (HCV) is a major public health concern, and the development of an effective HCV vaccine plays an important role in the effort to prevent new infections. Supramolecular co-assembly and co-presentation of the HCV envelope E1E2 heterodimer complex and core protein presents an attractive vaccine design strategy for achieving effective humoral and cellular immunity. With this objective, the two antigens were non-covalently assembled with an immunostimulant (TLR 7/8 agonist) into virus-mimicking polymer nanocomplexes (VMPNs) using a biodegradable synthetic polyphosphazene delivery vehicle. The resulting assemblies were characterized using dynamic light scattering and asymmetric flow field-flow fractionation methods and directly visualized in their vitrified state by cryogenic electron microscopy. The in vivo superiority of VMPNs over the individual components and an Alum-formulated vaccine manifests in higher neutralizing antibody titers, the promotion of a balanced IgG response, and the induction of a cellular immunity—CD4+ T cell responses to core proteins. The aqueous-based spontaneous co-assembly of antigens and immunopotentiating molecules enabled by a synthetic biodegradable carrier offers a simple and effective pathway to the development of polymer-based supramolecular nanovaccine systems.

## 1. Introduction

Hepatitis C virus (HCV) is a major public health concern and has chronically infected over 50 million people worldwide with nearly one million new infections every year [1]. HCV is the leading cause of liver cancer in North America, Europe, and Japan with high infection rates in Southeast Asia and other parts of the world [2,3]. Although highly effective direct-acting antivirals were introduced that can nearly eliminate the virus, the prevalence of HCV infection still remains high due to the limited availability to patients in low- and middle-income countries, and the inability to provide protection against reinfection [4,5,6]. This has prompted the World Health Organization (WHO) to announce a global hepatitis strategy, which aims to reduce new infections from all types of hepatitis viruses by 90% and associated deaths by 65% by 2030 [7]. The development of an effective HCV vaccine will play a major role in the achievement of this WHO mandate to address this major public health problem.

HCV is an enveloped, positive strand RNA virus that contains three structural proteins: core and two envelope glycoproteins, E1 and E2, and seven non-structural proteins [8,9]. The E1 and E2 glycoproteins are transmembrane proteins that form a membrane-anchored heterodimer complex, mbE1E2, which interacts with host cell receptors and is critical for viral entry into the cell. The E1E2 complex has been a prime subject of vaccine studies pursuing the induction of neutralizing antibodies [10]. More recently, a secreted E1E2 heterodimer (sE1E2) has been developed that maintains the natural conformational structure of mbE1E2 as a promising vaccine candidate [11,12,13].

A major challenge for HCV vaccine development is that the virus exhibits considerable genetic diversity, with eight known genotypes and more than one hundred subtypes identified thus far [14]. Given this diversity, the induction of broadly reactive immune responses that target genetically conserved regions of the viral genome will likely be required for protection against HCV. Data from both chimpanzee and human studies suggest that HCV-specific CD4+ and CD8+ T cells are important in the control of primary and secondary infections [10,15]. In addition, the clearance of HCV infection is also associated with the development of serum antibodies at an early time, capable of blocking the infection of multiple heterologous strains [16,17,18]. For these reasons, an effective vaccine must be capable of inducing both humoral and cellular responses. In view of this, the HCV core protein is more conserved across HCV genotypes compared to E1E2 and has been shown to be effective as an antigen in HCV prototype vaccines, providing strong cellular immune responses [19,20,21]. Therefore, vaccine designs combining the E1E2 heterodimer along with the HCV core protein are highly attractive in the context of inducing both B cell and T cell responses [22].

Virus-like particles (VLPs) represent a promising vaccine strategy due to their ability to mimic the structure of naturally occurring viruses without the transmission of infectious material. VLPs are categorized into enveloped and non-enveloped based on the presence or absence of lipid-based envelope proteins. Several non-enveloped VLP-based vaccines have been licensed for commercial use targeting hepatitis B virus and human-papillomavirus [23]. These vaccines tend to be more stable and easier to produce due to the absence of a lipid envelope protein. Envelope VLPs are more challenging in their design, treatment, and storage due to the importance of proper folding and the glycosylation of viral surface antigens. Several envelope VLPs are in clinical development including influenza virus, RSV, and SARS-Cov-2 [24]. It has been shown that both enveloped and non-enveloped VLPs are effective in stimulating both arms of the immune system, humoral and cellular responses. In reference to HCV, several expression systems have been used for HCV envelope VLP production including mammalian and insect cells by the co-expression of one or more of the HCV structural proteins such as E1E2 and core [25,26,27]. These studies showed promising results in preclinical model systems for the induction of potent humoral and cellular responses [27,28,29,30,31,32,33]. However, several limitations exist in the scale-up manufacturing of these envelope VLP vaccines including contamination with residual host cell or viral components, bioproduction yields, and/or the uniformity of the particles, thereby posing a challenge in VLP purification and vaccine development [26].

Due to these limitations, attempts to co-immobilize core and E1E2 proteins using synthetic delivery carriers have been undertaken in the past. For instance, “immune-stimulating complexes” (ISCOMs), which are composed of cholesterol, phospholipid, and saponin, showed significant potential. These components can form cage-like pentagonal dodecahedral structures (ISCOMATRIX) upon mixing, which were shown to be efficient in adsorbing core proteins via electrostatic interactions and eliciting cellular responses in macaques [34]. Further studies demonstrated that such core-adsorbed ISCOMATRIX vehicles can also serve as an efficient adjuvant for an E1E2 antigen in mice [34]. The described formulation approach, however, leads to a dramatic increase in the dimensions of complexes—from 40 nm for INSCOMATRIX to micrometer-sized particulates for antigen-loaded formulations. Although significant cellular responses were detected in macaques with ISCOMATRIX-core formulation, the exact mechanism remained unclear. Authors note that the internalization of micrometer-sized particulates by phagocytosis typically results in their delivery directly to lysosomes. This leads to antigen presentation of the Ag through the MHC class II pathway, which is primarily involved in humoral immune responses [34]. Furthermore, the evaluation of the ISCOMATRIX-HCV core vaccine in a Phase I clinical study showed no indication of dose response, with CD8^+^ T cell responses detected only in two of the eight participants receiving the highest vaccine dose [35].

Polymer-based nanoparticulates present an alternative approach to virus mimicry-based vaccine delivery [36,37,38,39,40,41,42,43,44]. Synthetic polyphosphazene macromolecules offer a compelling choice as water-soluble vehicles for the delivery of vaccine antigens. These macromolecules were shown to hydrolytically degrade at near physiological conditions with the release of physiologically benign compounds [45]. Their in vivo performance and safety were validated in multiple animal models and clinical studies [46,47]. The ability of these ionic polymers to spontaneously self-assemble with antigenic proteins in aqueous solutions is one of the key factors responsible for their immunopotentiating activity. The resulting nano-sized protein–polymer complexes are stable at near physiological conditions, retain excellent water solubility, and are capable of displaying antigenic molecules in a multimeric form [48,49,50,51]. Poly(dicarboxylatophenoxy)phosphazene (PCPP) is an advanced representative of this class of macromolecules, which demonstrated an excellent safety profile and dose-dependent immunoadjuvant effect in clinical trials.

In the present study, we introduced a novel approach in which we co-assembled two HCV antigens—sE1E2 and core protein, with a small molecule TLR7/8 agonist—R848 (resiquimod) [52,53,54,55], using a lightly PEGylated version of the PCPP macromolecule as an interconnecting delivery system. The resulting nano-sized complexes were directly visualized in their vitrified state by cryogenic electron microscopy (cryoEM), showed monomodal size distribution by dynamic light scattering (DLS), and confirmed to comprise antigenic and adjuvant components by asymmetric flow field-flow fractionation (AF4) method. We refer to these nano-sized polymer–protein complexes as virus-mimicking polymer nano-assemblies (VMPNs). In murine studies, we demonstrate that these VMPNs can be used as vaccine candidates that demonstrate in vivo superiority over the individual components by inducing higher neutralizing antibody titers, promote a balanced IgG class, and are capable of inducing CD4+ T cell responses to core proteins.

## 2. Materials and Methods

### 2.1. Plasmid Construction

For the expression sE1E2SZ.H445P protein, the human codon-optimized cDNA sequence was synthesized by GenScript and cloned into a pcDNA3.1(+) vector as described previously [12]. Plasmid expressing HCV core protein was kindly provided by Dr. Mariuzza, (Institute for Bioscience and Biotechnology Research, University of Maryland, Rockville, MD, USA).

### 2.2. Protein Expression and Purification

The transient expression of recombinant sE1E2SZ.H445P was performed in human Expi239 cells using the Expi293 Expression system by following manufacturer’s instructions (Thermo Fisher Scientific, Carlsbad, CA, USA). Briefly, Expi293 cells (ATCC, Manassas, VA, USA) were cultured in Expi293 Expression medium in the shaker incubator at 37 °C, with 120 rpm and 8% carbon dioxide. After cells reached a density of 2 × 10^6^ cells/mL, cells were cotransfected with sE1E2SZ.H445P and furin constructs (kindly provided by Dr. Yuxing Li, Institute for Bioscience and Biotechnology Research, University of Maryland, Rockville, MD, USA) at a 2:1 ratio. The culture supernatant of sE1E2SZ.H445P was harvested after 72 h, clarified by centrifugation and filtered through a 0.22 μM filter (Pall Corporation, Port Washington, NY, USA). Protein was then purified by sequential HisTrap Ni^2+^ -NTA and Superdex200 size-exclusion chromatography (Cytiva, Uppsala, Sweden) as described previously [11].

The HCV core protein was produced in *E.coli* cells, purified, and refolded from inclusion bodies. For expression, the HCV core construct was transformed into BL21(DE3) competent cells. A single colony from a fresh transformation was inoculated into 0.5 L of Luria–Bertani (LB) broth (Fisher Scientific, Hampton, NH, USA) medium containing 100 μg/mL of ampicillin. The culture was allowed to grow at 37 °C until it reached an OD_600_ of 0.6–0.7 and was induced with Isoprophyl β-D-1-thiogalactopyranoside (IPTG) (Gold Biotechnology, St. Louis, MO, USA) at a final concentration of 1 mM. After incubation for 3–3.5 h, the bacteria were harvested by centrifugation at 7000 rpm. The cell pellet was resuspended in 75–100 mL of 100 mM Tris-HCl pH 8, 150 mM NaCl, 1 mM EDTA, and 5% Triton X100 (Sigma-Aldrich, St. Louis, MO, USA) and sonicated for 5 min at ~50 kHz on ice. Inclusion bodies were centrifuged at 8000 rpm at 4 °C for 15 min. Next, the pellet was resuspended in 75–100 mL of 100 mM Tris-HCl pH 8, 150 mM NaCl, 1 mM EDTA, and further sonicated for 5 min at ~50 kHz on ice. Inclusion bodies were again centrifuged at 8000 rpm at 4 °C for 15 min. The remaining pellet was resuspended in 10–15 mL of 100 mM Tris-HCl pH 8, 8 M Urea (Fisher Scientific, Fairlawn, NJ, USA), and placed on a nutating mixer at 4 °C overnight. The dissolved inclusion bodies were centrifuged at 25,000 rpm for 1 h, and the remaining pellet was dissolved in 6 M Guanidinium chloride (Fisher Scientific, Fairlawn, NJ, USA) and centrifuged again. Solubilized inclusion bodies were refolded by adding dropwise to the refolding buffer 0.4 M Arginine-HCl (Sigma-Aldrich, St. Louis, MO, USA), 100 mM Tris-HCl pH 8.0, 3.7 mM Cystamine (Sigma-Aldrich, St. Louis, MO, USA), 6.6 mM Cysteamine (Sigma-Aldrich, St. Louis, MO, USA), and 5 mM EDTA and stirred at 4 °C for 72 hrs. The mixture was then concentrated and dialyzed in PBS buffer before loading onto a Superdex 200 increase 10/300 column (Cytiva, Uppsala, Sweden). The peak from the size exclusion column was loaded onto Pierce high-capacity endotoxin removal resin (Thermo Scientific, Rockford, IL, USA) to remove endotoxins. Refolded HCV core was aliquoted and stored at −80 °C.

Purified sE1E2SZ.H445P and HCV core proteins were separated by precast, 4–20% Mini-Protean TGX stain-free gels (Bio-Rad, Hercules, CA, USA) on a Mini-Protean Tetra cell electrophoresis instrument (Bio-Rad, Hercules, CA, USA). For Western blot (Supplementary Materials Figure S1), protein samples were transferred onto Trans-Blot Turbo Mini nitrocellulose membranes (Bio-Rad, Hercules, CA, USA) and probed with anti-HCV E2 mAb HCV.1 at 5 μg/mL, anti-HCV E1 mAb H-111 at 10 μg/mL followed by detection with a secondary goat anti-human IgG-HRP conjugate (Invitrogen) at a 1:10,000 dilution and the Western ECL substrate (Bio-Rad Laboratories, Inc., Hercules, CA, USA). The HCV core sample was probed with anti-HCV core C7-50 mAb (Invitrogen, Rockford, IL, USA) at 1μg/mL and detected with goat anti-mouse IgG-HRP conjugate (Abcam Inc., Waltham, MA, USA) at 1:10,000 dilution.

### 2.3. Synthesis and Characterization of PCPP-PEG

A copolymer of PCPP containing 2% (mol) of 5 kDa poly(ethylene glycol), PEG chains (PCPP-PEG) was synthesized using macromolecular substitution of polydichlorophosphazene (Supplementary Materials, Synthesis of PCPP-PEG and Figure S2a). Polymer structure and composition were confirmed by ^1^H NMR (400 MHz, D_2_O): δ [ppm] 7.3 (br, 2H, =CH_2_-); 6.5 (br, 2H, =CH_2_-]); 3.6 (br, 4H, -CH_2_-CH_2_-O) and ^31^P NMR (161.9 MHz, pH 14). δ [ppm]: −18.8 (Supplementary Materials Figure S2b). Molecular weight (510,000 g/mol) was determined by size exclusion chromatography using the Agilent 1260 Infinity II Binary LC instrument equipped with a G7117C diode array detector. A TSKgel GMPW column (Tosoh Bioscience, LLC, Tokyo, Japan), PEG molecular weight standards (American Polymer Standards Corporation, Mentor, OH, USA) and 0.1 × PBS with 10% acetonitrile (mobile phase) were employed. The chromatogram of PCPP-PEG along with molecular weight parameters and dispersity can be found in Supplementary Materials Figure S2c.

### 2.4. Preparation of Virus-Mimicking Polymer Nanocomplexes (VMPNs)

In a typical procedure, PCPP-PEG-based VMPNs were prepared as follows. PCPP-PEG (0.5 mL of 4 mg/mL in PBS) was formulated with R848 (0.5 mL 0.8 mg/mL in DI water, pH 6.2) to bind a cationic small-molecule TLR agonist with negatively charged carboxylic acid groups of the polymer. The resulting formulation was mixed with the solution of a core protein (1 mL of 0.02, 0.2 or 0.5 mg/mL), and then 2 mL of 0.25 mg/mL E1E2 protein were added under mixing by pipetting.

### 2.5. Physicochemical Characterization

Dynamic light scattering (DLS) analysis was carried out using a Malvern Zetasizer Nano ZS instrument equipped with version 7.10 Zetasizer Software (Malvern Instruments Ltd., Worcestershire, UK).

The asymmetric flow field-flow fractionation (AF4) analysis was conducted using the AF2000 AT instrument (Postnova Analytics Inc., Salt Lake City, UT, USA). The separation cartridge was equipped with a regenerated cellulose membrane (10 kDa molecular weight cutoff), and PBS (pH 7.4) was used as an eluent.

### 2.6. CryoEM Visualization of Complexes

An aliquot of polyphosphazene formulation (3.0 μL) was placed on holey carbon film transmission electron microscopy (TEM) grids (Electron Microscopy Sciences, Hatfield, PA, USA), and plasma treatment was performed using glow discharge PELCO EasiGlow (Ted Pella Inc., Redding, CA, USA). The grids were then blotted for 2 s at 12 °C twice using a Vitrobot system (Vitrobot Mark IV, FEI, Hillsboro, OR, USA) and vitrified in a liquid ethane. The samples were analyzed using a 200 kV Talos Arctica electron microscope equipped with an FEI Falcon3EC detector (FEI, Hillsboro, OR, USA). Visualization was carried out at 90 K (200 kV acceleration voltage). The results were collected with EPU software and processed in CryoSPARC 4.2.1 (Structura Biotechnology Inc., Toronto, ON, Canada). The brightness and contrast of the images were tuned using ImageJ software (Laboratory for Optical and Computational Instrumentation, University of Wisconsin, WI, USA) [56].

### 2.7. Immunization

Thirty-five female Balb/c mice aged 6–8 weeks (Jackson Labs, ME) were randomly distributed into seven groups of five mice each. We chose inbred Balb/c mice because we expect a more uniform immune response than, e.g., CD1 outbred mice, thereby giving us a better opportunity to detect differences between groups. The group size was chosen based on calculations described previously [57]. The mice were immunized with PCPP-PEG (50 µg)-R848 (20 µg)-based VMPN formulations containing 25 µg of HCV envelope-sE1mE2.SZ_H445P antigen alone (Group 1) or a combination of 25 µg sE1mE2.SZ_H445P and HCV core protein, with the core protein included at 1 µg (Group 2), 10 µg (Group 3), or 25 µg (Group 4), respectively. The mice in the remaining groups received 25 µg sE1mE2.SZ_H445P and 10 µg core protein formulated in PCPP-PEG alone (50 µg PCPP-PEG, Group 5) or Alum (35 µg Alum, Group 6) or no adjuvant (control Group 7), respectively (Table 1). Thus, groups 1–4 provide an analysis of the dose-dependence of HCV core effects in the context of constant sE1mE2.SZ_H445P and the PCPP-PEG+R848 adjuvant. Groups 5–7 provide three different controls: one group that is equivalent to group 3, but lacking R848; one group that is equivalent to group 3 but uses Alum instead of PCPP-PEG+R848 as the adjuvant; and one group that contains the same antigens as group 3 but no adjuvant. The mice were immunized by the intraperitoneal route. The immunization regimen comprised prime immunization on day 0, followed by boosts on days 14, 28, and day 42, respectively. Blood samples were collected on days 0, 14, 28, 42, and 56, processed for serum by centrifugation, and stored at −80 °C. For memory T cell assays, mice were given an additional boost on Day 136 and then euthanized 7 days later for analysis. Mice were observed daily for mortality and signs of morbidity and weighed biweekly. One mouse (#15) from Group 3 was found dead overnight on day 15 following boost 1, and this was deemed to have resulted from injury from intraperitoneal immunization. Post immunization for all mice in the study, there was no signs of morbidity, hunched posture, ruffled fur, lethargy, diarrhea, and/or loss of >20% body weight associated with the administration of vaccine formulations.

### 2.8. Enzyme-Linked Immunosorbent Assay (ELISA)

ELISAs were performed in 96-well plates (MaxiSorp, Thermo Fisher Scientific, Waltham, MA, USA) coated overnight with 5 μg/mL of Galanthus Nivalis Lectin (Vector Laboratories, Newark, CA) at 4 °C. Plates were then washed with phosphate-buffered saline (PBS) containing 0.05% Tween 20 and coated with sE1E2SZ.H445P antigen (200 ng per well) at 4 °C overnight. Plates were then washed and blocked with blocking buffer 1xPBS, 2% dry milk, and 5% fetal bovine serum (FBS, GIBCO, Grand Island, NY, USA) for 1 h at room temperature. Serially diluted serum samples were added to the plates and incubated for 1 h. The binding of HCV E1E2-specific antibodies was detected by a 1:5000 dilution of HRP-conjugated goat anti-mouse IgG (H+L), IgG1, and IgG2a secondary antibody (Southern Biotech, Homewood, AL, USA) with TMB substrates (Bio-Rad, Hercules, CA, USA). Absorbance values were read at 450 nm (SpectraMax M3 microplate reader, Molecular Devices, San Jose, CA, USA) and used to determine endpoint titers, which were calculated by curve fitting using GraphPad Prism software version 10 (Dotatics, San Diego, CA, USA) and defined as four times the highest absorbance value of preimmune sera.

### 2.9. HCV Pseudoparticles (HCVpp) Neutralization Assay

Huh7, a human hepatoma cell line (European Collection of Authenticated Cell Cultures (ECACC), Salisbury, UK), was grown and maintained in a DMEM media supplemented with 10% heat-inactivated FBS (GIBCO) and 5% Penicillin–Streptomycin (10,000 U/mL) (Thermo Fisher Scientific, Waltham, MA, USA) and used as the target cell line for neutralization assays [58]. Huh7 cells were seeded onto 96-well plates at a density of 1 × 10^4^ per well to test sera for neutralization. The following morning, the pseudoparticles (HCVpp) were incubated with defined serial dilutions of heat-inactivated serum for 1 h at 37 °C and added to each well. Plates were incubated in a CO_2_ incubator at 37 °C for 5 to 6 h, after which the mixtures were replaced with fresh medium and then incubated for 72 h. After incubation, 100 μL of Bright-Glo (Promega, Fitchburg, WI, USA) was added to each well for 2 min at an ambient temperature. The luciferase activity was measured using a FLUOstar Omega plate reader (BMG Labtech, Ortenberg, Germany) and stored with the MARS software (Friday Harbor, WA, USA). The ID_50_ was calculated as the serum dilution that caused a 50% reduction in relative light units (RLUs) compared with HCVpp in the control wells. All values were calculated using a dose–response curve fit with nonlinear regression in GraphPad Prism. All experiments using HCVpps were performed under biosafety level 2 conditions.

### 2.10. Intracellular Staining for Cytokines and Low Cytometry Analysis

Single-cell suspensions from the lymph nodes and spleens of immunized animals were incubated with specific antigen or 100 ng/mL of Phorbol 12-myristate 13-acetate (PMA; Millipore Sigma, St. Louis, MO, USA) and 1 µg/mL of Ionomycin (Millipore Sigma, MO, USA) in complete RPMI (Thermo Fisher Scientific, Waltham, MA, USA) for 2 h at 37 °C. At this time, cells were treated with 10 µg/mL of Brefeldin A (eBioscience, San Diego, CA, USA) for 4 h at 37 °C. For staining, Fc receptor blocking (using a cocktail of 2.4G2 (BioXcell, Hayward, CA, USA) and chrompure IgG (Jackson labs, Bar Harbor, ME, USA) was performed for 15 min at 4 °C in a FACS buffer (1X PBS supplemented with 0.2% BSA, 0.01% azide). Surface staining was performed for 30 min at 4 °C in a FACS buffer using the indicated antibodies. Cells were washed with a FACS buffer, fixed and permeabilized in a BD Cytofix/Cytoperm solution (BD Biosciences, Milpitas, CA, USA) for 20 min at 4 °C, followed by an overnight incubation at 4 °C in 1X Fixation/Permeabilization Solution (eBioscience, San Diego, CA, USA). Intracellular staining was performed using the indicated antibodies in 1X eBioscience Permeabilization Buffer for 1 h at 4 °C. Stained cells were washed twice with 1X Permeabilization Buffer (eBioscience, San Diego, CA, USA) followed by another two washes with a FACS buffer. Cells were analyzed on the BD LSR-II cytometer and all data were analyzed using FlowJo (TreeStar Inc., Ashland, OR, USA).

### 2.11. Statistical Analysis

The nonparametric Kruskal–Wallis test with Dunn’s multiple comparisons test was used for the statistical analysis of differences among group % neutralizations (*p* < 0.05 was considered significant). All statistical analyses were conducted using GraphPad Prism software, version 10 (Dotatics, San Diego, CA, USA).

## 3. Results

### 3.1. Macromolecular Design of a Polyphosphazene Delivery Vehicle and Its Optimization

**Figure 1:**
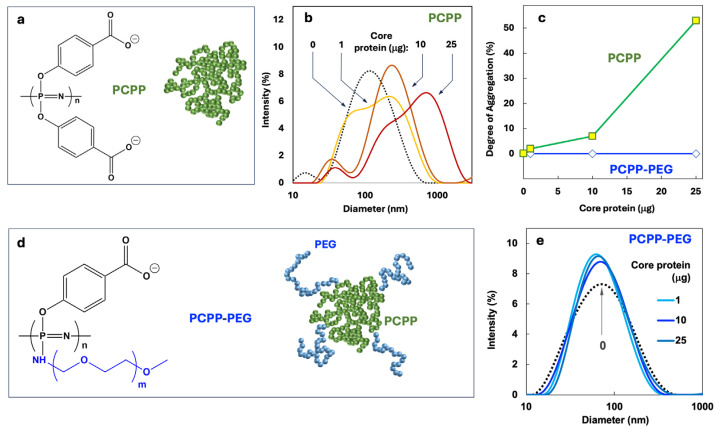
Selection of PCPP-PEG as a polyphosphazene delivery vehicle for preparing VMPNs is made based on the stability of its complexes with HCV core antigen. (**a**) Chemical structure and schematic presentation of PCPP, (**b**) DLS profiles of PCPP and its complexes with core protein, (**c**) degree of aggregation determined by DLS as a function of core protein concentration for PCPP and PCPP-PEG, (**d**) chemical structure and schematic presentation of PCPP-PEG, and (**e**) DLS profiles of PCPP-PEG and its complexes with core protein (25 µg E1E2, 50 µg PCPP or PCPP-PEG, PBS, pH 7.4).

From the biophysical standpoint, the optimal design of a polyphosphazene delivery vehicle, which is capable of carrying multiple proteins and small molecules, has to satisfy at least two main criteria. First, the system should enable nano-scale dimensions of the resulting assemblies and prevent uncontrolled agglomeration. Second, sufficient avidity between components should be provided. Initially, the formulation was undertaken using a clinical stage polyphosphazene immunoadjuvant—PCPP (Figure 1a). However, the DLS study of the molecular interactions of this polymer with the core protein revealed a severe dose-dependent agglomeration in the system (Figure 1b,c). This problem was alleviated through the synthesis of a structural analog of PCPP, which contains 2% (mol) of graft PEG chains (Figure 1d). Such structural modification of the polymer using short chains of hydrophilic PEG allowed for a complete prevention of agglomeration (Figure 1c,e). Furthermore, PCPP-PEG copolymer showed superior resistance to agglomeration caused by acidic and high ionic strength environments (Supplementary Materials Figure S3). No agglomeration of PCPP-PEG was detected upon the addition of the E1E2 antigen or R848 to PCPP-PEG in doses intended for in vivo experiments.

### 3.2. Assembly of VMPNs Containing Ternary E1E2—Core Protein—TLR7/8 Agonist

**Figure 2:**
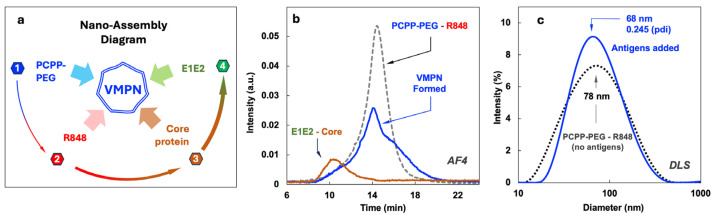
Preparation and characterization of PCPP-PEG-based VMPNs containing R848 and HCV antigens. (**a**) Schematic diagram of VMPN nano-assembly showing sequential steps of component addition, (**b**) AF4 profiles showing protein–polymer binding, and (**c**) DLS profiles of VMPNs contrasted with protein-free polymer (z-average diameters are shown, pdi—polydispersity index).

The nano-assembly of four-component VMPNs was conducted through the sequential addition of R848, E1E2, and core protein to the aqueous solution of PCPP-PEG (Figure 2a). PCPP-PEG, which contains negatively charged carboxylic acid moieties, was first modified with positively charged small molecule, R848, to afford its binding to the polymer as a counterion (Supplementary Materials Figure S4), as described previously [59]. The binding of a sequentially added core protein and E1E2 to a PCPP-PEG scaffold modified with R848 was demonstrated by AF4 analysis. This method allows for the separation and detection of analyte components based on their molecular and supramolecular sizes [60,61] and has been previously successfully applied to polyphosphazene systems [49,62]. Figure 2b shows AF4 fractogram of VMPNs (solid line at approximately, peak at 14 min) contrasted with the PCEP-PEG—R848 system (dashed line) and a mixture of E1E2—core proteins (peak at 10 min). The binding of proteins to the polymer scaffold is demonstrated by a complete disappearance of protein peaks (10 min) in the VMPN and change in a peak profile compared to PCPP-PEG—R848 (dashed line, peak at 14 min). The latter indicates a modified character of interactions with the analytical membrane of AF4, which is caused by the binding of proteins and the formation of VMPN. The hydrodynamic diameter of VMPN was determined by DLS and roughly corresponded to the dimensions of the protein-free polyphosphazene scaffold (about 70 nm). The formulation shows unimodal distribution and is completely free of agglomerates. The z-potential of all PCPP-PEG formulations maintained negative values, shifting from −8.07 mV for the polymer to −6.13 mV for PCPP-PEG with R848 counterions and −5.64 mV for VMPNs containing antigens.

### 3.3. Direct Visualization of VMPNs by cryoEM

**Figure 3:**
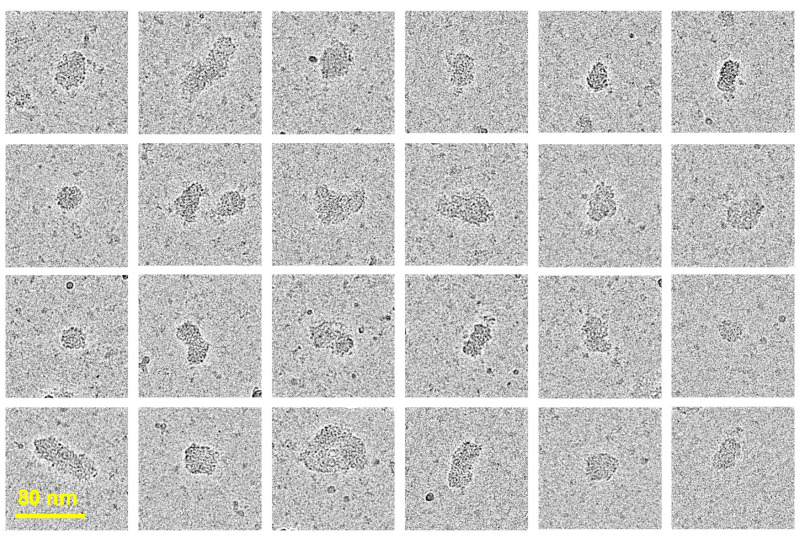
CryoEM images of PCPP-PEG-based VMPNs containing R848 and HCV antigens in their vitrified state (50 mM phosphate buffer, pH 7.4).

Cryogenic electron microscopy (cryo-EM) is an advanced technique used for visualizing biomacromolecules, such as proteins. It provides images of biomolecules embedded in vitreous, glass-like ice, revealing the real-space architecture of macromolecules and their nanoassemblies in their native environment [63,64,65,66]. Although the visualization of synthetic polymer chains has been challenging due to the low electron optical contrast of the backbone and their inherent flexibility [67,68,69], the technique was proven to be successful for the analysis of their hierarchical structures, such as micellar assemblies [70,71,72,73]. CryoEM images of E1E2 and core protein-loaded VMPNs are shown in Figure 3. The VMPN assemblies can be described as compact spherical nanocomplexes of somewhat irregular shape. The shape, diameter, and size distribution of VMPNs are reminiscent of HCV virions visualized using the same technique [74] and are strikingly different from the single chains of PCPP macromolecules [48]. The overall dimensions of VMPNs seen from the cryoEM images are in line with those determined by DLS (approximately 60–70 nm, Figure 2c) and are only marginally larger than those of HCV virions (approximately 50 nm) [74].

### 3.4. Immunization Studies

The immunogenicity of VMPNs was evaluated in Balb/c mice. Three animal groups were immunized with VMPNs containing the polyphosphazene–R848 system (PPZ-R) assembled with E1E2 (25 μg) and various doses of core protein (1, 10, and 25 μg) (Table 1, groups 2–4). These groups were compared with animals that received VMPNs without a core protein and R848-free VMPNs containing 10 μg of core protein (groups 1 and 5, respectively). The results were also benchmarked against those in animals immunized with a mixture of E1E2–core proteins adsorbed on Alum or formulated without a delivery vehicle (groups 6 and 7, respectively). The immunization schedule included prime and three boosts.

**Table 1:** Immunization groups.

Group	sE1E2-H445P (µg)	Core (µg)	Vehicle			Reference
			PCPP-PEG (µg)	R848 (µg)	Alum (µg)	
1	25	-	100	20	-	PPZ-R (sE1E2)
2	25	1	100	20	-	PPZ-R (sE1E2-C1)
3	25	10	100	20	-	PPZ-R (sE1E2-C10)
4	25	25	100	20	-	PPZ-R (sE1E2-C25)
5	25	10	100	20	-	PPZ (sE1E2-C10)
6	25	10	-	-	35	Alum (sE1E2-C10)
7	25	10	-	-	-	(sE1E2-C10)

### 3.5. Evaluation of Serological Responses and Homologous and Heterologous Neutralization

**Figure 4:**
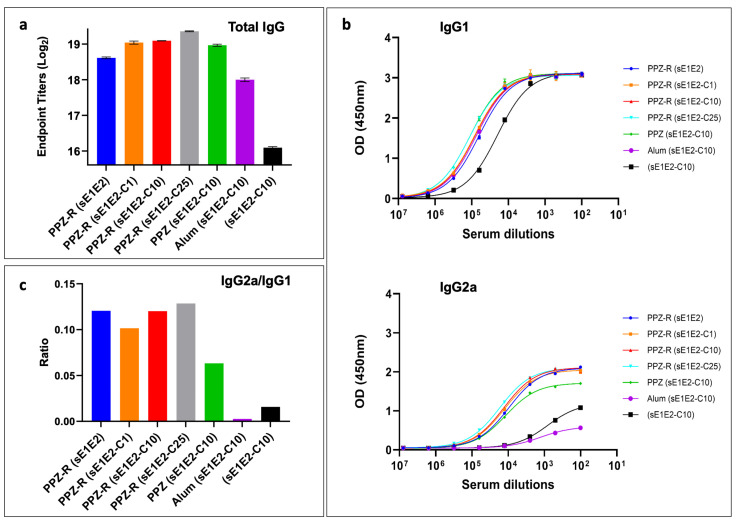
Immunogenicity assessment of PPZ-based and Alum formulations by ELISA of antibodies induced in immunized mice at day 56 after immunization. (**a**) Total IgG titers, (**b**) IgG1 and IgG2a titers, and (**c**) the ratio of IgG2a to IgG1 for each immunogen. Pooled sera from the indicated groups were analyzed. Endpoint titers were calculated by curve fitting in GraphPad software (version 10) with endpoint optical density defined as four times the highest absorbance value of day 0 sera.

Figure 4a shows that total IgG titers are influenced by both the addition of R848 and core protein, with both leading to an increase in overall titers. Moreover, all of the PPZ formulations elicit higher titers than antigens formulated with Alum or without any adjuvant. The isotype-specific analysis of IgG1 and IgG2a titers shows that, while the IgG1 titers do not vary appreciably between groups (Figure 4b, upper panel), there is a marked disparity in the IgG2a titers (Figure 4b, lower panel), which act as a proxy measure for the strength of the cellular immune response. Among these groups, the differences that achieve statistical significance in an uncorrected pairwise Kruskal–Wallis test are PPZ-R(sE1E2-C10) versus Alum(sE1E2-C10), PPZ-R(sE1E2-C25) versus sE1E2-C10 (*p* < 0.05), and PPZ-R(sE1E2-C25) vs. Alum(sE1E2-C10) (*p* < 0.01).

**Figure 5:**
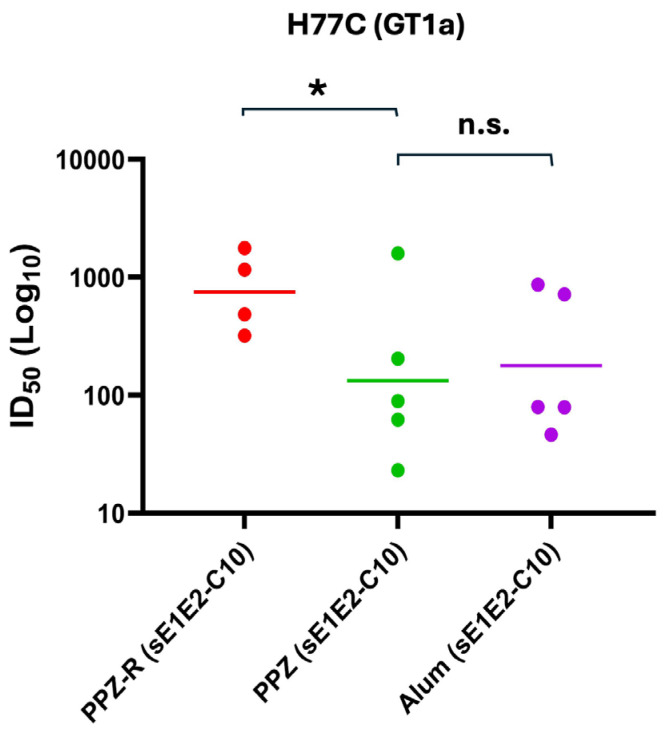
Potency of neutralization of individual mouse sera against H77C (GT1a) comparing PPZ and Alum-based formulations. Individual immunized mice sera were assessed for neutralization activities at day 56 and day 0. Serum dilutions were performed as three-fold dilutions starting at 1:150 for HCVpp neutralization. The experiment was performed in duplicate, and the geometric mean of each group is denoted. *p* values were calculated using the Kruskal–Wallis analysis of variance with Dunn’s multiple comparison test, and significant *p* values are shown (* *p* < 0.05, n.s. *p* > 0.5).

The presence of core protein did not have a dose-dependent effect on IgG2a levels, but the inclusion of R848 did appear to enhance IgG2a levels. Remarkably, the Alum formulation barely elicited any IgG2a at all, and in fact performed worse in this regard than the no adjuvant group (Figure 4b, lower panel). As a result, the immune response was skewed such that for the Alum group, the IgG2a/IgG1 ratio is near zero, whereas the PPZ groups, and, in particular, the PPZ-R groups, show a much more balanced immune response with ratios of 0.1 or higher (Figure 4c). Based on these data, we analyzed the ability of mice immunized with sE1E2 and 10 μg of core protein to neutralize HCV pseudoviruses from the homologous GT1a (H77) strain (Figure 5 and Figure S6) and the heterologous strains (Table 2 and Figure S5). Three heterologous strains were chosen, one from GT1b (UKNP1.18.1), one from GT2a (J6), and one from GT2b (UKNP2.5.1). The PPZ-R group performs the best in neutralizing the homologous pseudovirus, with a mean ID_50_ that is more than three times that of the other two groups. This difference achieves statistical significance for PPZ-R versus PPZ, but not versus Alum (likely due to data spread). Heterologous neutralization was generally weak, and the data are inconclusive, with each group neutralizing one of the three heterologous pseudoviruses better than the other two groups.

### 3.6. Evaluation of Memory VMPN T-Cell Responses to sE1E2 and Core Antigens

**Figure 6:**
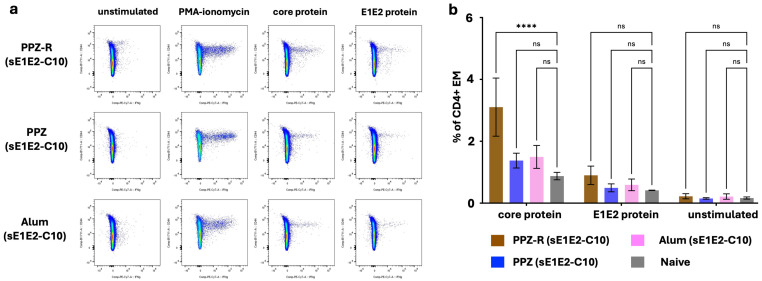
T cell responses to PPZ-R-adjuvanted HCV antigens as compared to those from PPZ or Alum immunizations. (**a**) Representative Intracellular Cytokine Staining FACS plots showing PPZ-R, PPZ, or Alum-vaccinated mice, stimulated as labeled on each column. Plots are gated on CD4+ T cells and (**b**) quantitation of the total percentage of IFNγ-producing CD4+ CD44-hi T cells in each condition, after stimulation with core or E1E2 recombinant proteins (**** *p* < 0.0001, ns *p* > 0.5).

Antigen-specific memory T cells primed by a vaccine must persist for a long time in animals to provide enhanced protection during a subsequent rechallenge. Typically, effector T cells (marked by higher levels of the surface marker CD44) are formed in the first weeks after immunization, but the persistence of antigen-specific T cells with such a phenotype beyond the 3–4 months of challenge is evidence of the generation of durable immunological memory. Accordingly, to evaluate the presence of long-term memory T cell responses generated by the VMPNs in mice, we further boosted immunized mice at Day 136 and isolated single-cell suspensions 7 days later. Effector cytokine production in an antigen-specific manner is a strong correlate of such memory T cells [75]. The data in Figure 6 compare the responses of isolated splenocytes by measuring intracellular IFNγ upon restimulation with E1E2 or core proteins (with PMA/Ionomycin used as a positive control polyclonal stimulator). Cells were analyzed by gating on the live CD4+ population (gating strategy discussed in Supplementary Materials Figure S7). CD4 T cells from the PPZ-R-vaccinated mice, re-stimulated with core protein, show a significant increase in the proportion of IFNγ producers within the CD44-hi (effector/memory phenotype) subset compared to those from PPZ or Alum immunizations. At this time-point, no significant response to E1E2 proteins was observed; in previous studies, lymphoproliferative responses tended to be higher for core than for E1E2 [30].

## 4. Discussion

We previously reported the supramolecular assembly of PCPP with the toll-like receptor 7/8 agonist, R848, that could significantly enhance immune stimulation in vitro and in vivo to the HCV E2 protein [59]. In the present study, we expanded on this assembly approach and analyzed the antigenic compositions and immunogenicity of VMPNs comprising the HCV sE1E2 envelope heterodimer and core protein. The secreted sE1E2 heterodimer is a molecular mimic of the membrane-associated form that can be uniformly produced and shown to induce robust, cross-neutralizing antibody responses in mice [12]. In contrast, the purification of membrane-associated E1E2 results in heterogeneous agglomerates at low yield that could contain host cell proteins, thereby limiting its potential as a vaccine antigen [13]. For these reasons, we favor the production and purification of secreted sE1E2 in mammalian cells that is more easily controlled and free of host cell components that pose potential regulatory challenges. The core protein is the most highly conserved HCV protein among the different viral genotypes, and cellular immune responses to core protein have been associated with improved responses to antiviral treatment and disease outcome. Since core contains no post-translation modifications, it can be readily produced in *E. coli,* which has many advantages including low-cost production and the simplicity of scaling up. Therefore, we view our synthetic VMPN incorporating sE1E2 and core as a chemically, well-defined envelope VLP-type platform designed to elicit both humoral and cellular immunity to further advance the development of an HCV vaccine.

The efficacy of a vaccine depends not only on the antigen components but also is increasingly reliant on the adjuvants used to stimulate the immune system. Adjuvants are typically classified according to their physicochemical properties, biochemical characteristics, and mechanisms of action. They can be further divided into two main categories: delivery systems and immune stimulators. Research efforts on the development of HCV vaccine candidates have largely focused on the search for potent immunoadjuvants in which side-by-side comparisons are relatively uncommon [76]. Alum (Alhydrogel), along with various emulsion-based systems such as MF59 [77], remain the most popular choices in such studies. Polyphosphazene adjuvants used in the present study combine delivery vehicle characteristics, which are realized through the association of polymer with the antigen, with the capability of inducing a local proinflammatory response leading to the recruitment of immune cells to the site of injection [46].

PCPP is a clinical stage polyphosphazene macromolecule that has demonstrated promising immunoadjuvant properties in clinical settings [78,79,80,81]. Similarly to another ionic adjuvant, Alum, PCPP has been shown to bind (adsorb) antigenic macromolecules non-covalently to realize its immunopotentiating activity [82,83,84]. However, in contrast to Alum, which can only exist in a three-dimensional physical state as a hydrogel [85,86,87], PCPP is inherently water soluble. This important physical feature is also maintained for the majority of its nano-scale complexes with antigenic proteins, which makes PCPP highly attractive from the formulation standpoint [46]. Yet, our attempts to utilize PCPP as a carrier for all three payload molecules—sE1E2 and core proteins along with R848 TLR7/8 agonist—resulted in the undesirable and uncontrolled phase separation (Figure 1b). To alleviate this issue, we synthesized a new polyphosphazene derivative of PCPP, which contained 2% (mol) of relatively short (5 kDa) graft poly(ethylene glycol) (PEG) chains. The presence of a small PEG content in PCPP did not detrimentally affect the ability of the polymer to efficiently bind antigenic and immunoadjuvant components, which was proven by AF4 experiments (Figure 2b). However, such light PEGylation allowed for a dramatically improved solution behavior of the resulting nanocomplexes (Figure 2c). Moreover, immunization experiments confirmed that a new PCPP-PEG macromolecule was effective in facilitating immune responses in animals. It can be expected that PCPP-PEG can be a potent alternative to PCPP in cases when antigen–polymer interactions are sufficiently strong to cause charge neutralization and undesirable phase separation.

The simplicity of the supramolecular assembly of all payload components in aqueous solutions is extremely attractive from the formulation standpoint as it basically involves the sequential addition of the components. It links all formulation components together and results in monomodal size distribution in a nano-scale range. AF4 and DLS methods allow full in-process control of the assembly (Figure 2).

The CryoEM visualization approach facilitated by the high-contrast properties of the polyphosphazene backbone [48] reveals the morphological features of VMPN assemblies. As mentioned above, the shape and size characteristics of VMPNs resemble actual HCV virions when imaged in their vitrified state [74]. It is also important to note that the co-immobilization of the TLR7/8 agonist on VMPNs is critical for achieving both high neutralization titers and cellular responses (Figure 5 and Figure 6). As R848 was noted to be somewhat ineffective as vaccine adjuvant in vivo due to its rapid dissociation from the antigen [52,53,54,55], the effect can be attributed to its complexation with the polyphosphazene carrier (Supplementary Materials Figure S4) [59].

The in vivo evaluation of PCPP-PEG complexed with R848 (referred to as PPZ-R) and formulated with sE1E2 in the absence and presence of increasing amounts of core (1, 10, and 25 μg) was performed in Balb/c mice. These formulations were compared to Alhydrogel (Alum) and no adjuvant controls (Table 1). As shown in Figure 4a, the increasing amounts of core in the VMPN assemblies in the presence of R848 (PPZ-R (sE1E2-C1), PPZ-R (sE1E2-C10), and PPZ-R (sE1E2-C25)) resulted in an elevation in the anti-E1E2 IgG endpoint titers. Although there were no apparent significant differences among these groups, it is noteworthy that increasing the amount of core did not disrupt the biophysical properties of the PPZ-R-assembled VMPNs as presented to the immune system. Moreover, this is also reflected in the IgG1 titers (Figure 2b, upper panel) in which there is no significant difference among these groups. Lastly, the PPZ-R group performed the best in neutralizing the homologous pseudovirus (H77C, Figure 5) in which there is a significant difference in the mean ID_50_ that is more than three times that of the other two groups. This difference is likely due to the elevated total IgG response, but there was also a more uniform clustering of ID_50_ values around the mean in mice immunized with the PPZ-R adjuvant versus the PPZ and Alum groups. The observed variability in the immune response is common in animal studies, and our group has observed this phenomenon in the past using different HCV antigens such as mbE1E2, sE1E2, and the E2 ectodomain (sE2) [12,59,88]. The variability in neutralization potency, i.e., as judged by the spread in ID_50_ values, occurs for both homologous and heterologous neutralization. The basis of this variability is unclear, but there is some unpublished evidence to suggest that heterogeneity in the post-translational modifications of the antigen imparted by standard biomanufacturing systems plays a role. In addition, our conclusions are limited by weak cross-neutralization, so the enhanced neutralization effect observed for the homologous strain is not observed for the heterologous strains. However, given the low ID_50_ values for the heterologous strains tested, firm conclusions cannot be drawn for the effect of PPZ-R and HCV core on heterologous neutralization.

Effective vaccines against infectious agents require the elicitation of both humoral and cellular responses to engender protective immunity, along with persistent memory responses [89]. This has led to the design of tailored immunoadjuvant systems that can drive specific alterations of the immune response according to the specific chemical pattern of a pathogen, e.g., double-stranded RNA of viral origin for TLR3, bacterial lipopolysaccharide (LPS) for TLR4, singled-stranded RNA for TLR7/8, unmethylated CpG motifs found in bacterial DNA or viruses for TRL9, and so forth [90]. To that end, achieving a functionally appropriate type of immunity, such as T helper 1 (Th1)-mediated immunity, is highly desirable. We have shown previously that the supramolecular assembly of the R848 TRL7/8 agonist with PCPP drive can drive and modulate IgG1 and IgG2 isotypes in mice that are commonly used on the surrogate markers of Th2 and Th1 responses, respectively [59]. In this study, we show that the inclusion of R848 (PPZ-R) significantly enhances the IgG2a responses (Figure 2b, lower panel), and the resulting IgG2a/IgG1 ratio was significantly higher than in the absence of R848. Moreover, the IgG2a/IgG1 ratio was near zero for the Alum group in which the IgG2a response was negligible. This is further supported by the ability to increase the generation of memory T cells, a highly desirable feature of potent vaccine adjuvants. CD4 T cells play a critical role in the maintenance of functional CD8 responses as well as the formation of memory B cells [91]. Their function is typically evaluated by the production of cytokines, such as gamma interferon (IFN-γ) and tumor necrosis factor alpha (TNF-α), with multiple cytokine-producing cells considered to be superior. As shown in Figure 6, CD4 T cells from the PPZ-R-vaccinated mice, re-stimulated with core protein, show a significant increase in the proportion of IFNγ producers within the CD44-hi (memory phenotype) cell population compared to those from PPZ or Alum immunizations. Although we did not see a similar response to sE1E2 under these conditions, it has been shown that the E1E2 protein exhibits immunosuppressive properties, thereby making it a more challenging immunogen [92,93]. These results demonstrate the generation of antigen-specific memory responses in mice immunized with PPZ-R-adjuvanted antigens in the VMPN assemblies, and correlate with data on IgG isotype profiles confirming that the addition of PPZ-R favors Th1 responses. Overall, demonstrations that synthetic VMPNs can be assembled via simple and highly controlled formulation approaches and display potent immunostimulating activity in vivo, suggest that these bifunctional polymer-based supramolecular constructs can serve as a viable approach for parenteral vaccine applications. Given the simplicity of the formulation approach and the clinical history of PCPP adjuvant, we believe this approach possibly offers significant advantages for scalability, stability, and regulatory approval as compared to other envelope VLP vaccine approaches.

## Figures and Tables

**Table 2:** Homologous and heterologous neutralization serum ID_50_ values.

HCVpp *	PPZ-R (sE1E2-C10)	PPZ (sE1E2-C10)	Alum (sE1E2-C10)
GT1a	587.9	173.3	184.4
GT1b	36.7	65.6	72.4
GT2a	125.9	113.7	102.0
GT2b	73.4	109.9	63.4

* Please see Section 3.5 for more details on genotypes tested here.

## Data Availability

The original contributions presented in this study are included in the article/supplementary material. Further inquiries can be directed to the corresponding authors.

## Supplementary Materials

The following supporting information can be downloaded at: https://www.mdpi.com/article/10.3390/jfb16010034/s1, see [94]. Table S1: Binding affinities HCV envelope antigens using human mAbs. Figure S1: Characterization of sE1E2.SZ-H445P and HCV core antigens. Figure S2: Synthesis of PCPP-PEG. Figure S3: DLS studies demonstrating superior environmental stability of PCPP-PEG over PCPP. Figure S4: Ionic binding of R848 to a PCPP-PEG side chain. Figure S5: Breadth of neutralization against 4 core genotypes with HCVpp. Figure S6: Breadth of neutralization of individual mouse sera against H77C (GT1a). Figure S7: Gating strategy.

## References

[1] WHO Hepatitis C Key Facts. https://www.who.int/news-room/fact-sheets/detail/hepatitis-c.

[2] Fiehn F., Beisel C., Binder M. (2024). Hepatitis C virus and hepatocellular carcinoma: Carcinogenesis in the era of direct-acting antivirals. Curr. Opin. Virol..

[3] Costa G.L., Sautto G.A. (2024). Exploring T-Cell Immunity to Hepatitis C Virus: Insights from Different Vaccine and Antigen Presentation Strategies. Vaccines.

[4] Yukawa Y., Tamori A., Iio E., Ogawa S., Yoshida K., Uchida-Kobayashi S., Enomoto M., Tanaka Y., Kawada N. (2019). Hepatitis C virus recurrence in two patients who achieved sustained viral response with interferon-free direct-acting antiviral therapy: Reinfection or relapse?. Clin. J. Gastroenterol..

[5] Berenguer J., Gil-Martin A., Jarrin I., Montes M.L., Dominguez L., Aldamiz-Echevarria T., Tellez M.J., Santos I., Troya J., Losa J.E. (2019). Reinfection by hepatitis C virus following effective all-oral direct-acting antiviral drug therapy in HIV/hepatitis C virus coinfected individuals. AIDS.

[6] Rossi C., Butt Z.A., Wong S., Buxton J.A., Islam N., Yu A., Darvishian M., Gilbert M., Wong J., Chapinal N. (2018). Hepatitis C virus reinfection after successful treatment with direct-acting antiviral therapy in a large population-based cohort. J. Hepatol..

[7] WHO (2024). Global Hepatitis Report 2024.

[8] Bartenschlager R., Ahlborn-Laake L., Yasargil K., Mous J., Jacobsen H. (1995). Substrate determinants for cleavage in cis and in trans by the hepatitis C virus NS3 proteinase. J. Virol..

[9] Grakoui A., Wychowski C., Lin C., Feinstone S.M., Rice C.M. (1993). Expression and identification of hepatitis C virus polyprotein cleavage products. J. Virol..

[10] Cox A.L. (2020). Challenges and Promise of a Hepatitis C Virus Vaccine. Cold Spring Harb. Perspect. Med..

[11] Guest J.D., Wang R., Elkholy K.H., Chagas A., Chao K.L., Cleveland T.E.t., Kim Y.C., Keck Z.Y., Marin A., Yunus A.S. (2021). Design of a native-like secreted form of the hepatitis C virus E1E2 heterodimer. Proc. Natl. Acad. Sci. USA.

[12] Wang R., Suzuki S., Guest J.D., Heller B., Almeda M., Andrianov A.K., Marin A., Mariuzza R.A., Keck Z.Y., Foung S.K.H. (2022). Induction of broadly neutralizing antibodies using a secreted form of the hepatitis C virus E1E2 heterodimer as a vaccine candidate. Proc. Natl. Acad. Sci. USA.

[13] Toth E.A., Chagas A., Pierce B.G., Fuerst T.R. (2021). Structural and Biophysical Characterization of the HCV E1E2 Heterodimer for Vaccine Development. Viruses.

[14] Borgia S.M., Hedskog C., Parhy B., Hyland R.H., Stamm L.M., Brainard D.M., Subramanian M.G., McHutchison J.G., Mo H., Svarovskaia E. (2018). Identification of a novel hepatitis C virus genotype from Punjab, India: Expanding classification of hepatitis C virus into 8 genotypes. J. Infect. Dis..

[15] Duggal P., Thio C.L., Wojcik G.L., Goedert J.J., Mangia A., Latanich R., Kim A.Y., Lauer G.M., Chung R.T., Peters M.G. (2013). Genome-wide association study of spontaneous resolution of hepatitis C virus infection: Data from multiple cohorts. Ann. Intern. Med..

[16] Pestka J.M., Zeisel M.B., Blaser E., Schurmann P., Bartosch B., Cosset F.L., Patel A.H., Meisel H., Baumert J., Viazov S. (2007). Rapid induction of virus-neutralizing antibodies and viral clearance in a single-source outbreak of hepatitis C. Proc. Natl. Acad. Sci. USA.

[17] Osburn W.O., Snider A.E., Wells B.L., Latanich R., Bailey J.R., Thomas D.L., Cox A.L., Ray S.C. (2014). Clearance of hepatitis C infection is associated with the early appearance of broad neutralizing antibody responses. Hepatology.

[18] Logvinoff C., Major M.E., Oldach D., Heyward S., Talal A., Balfe P., Feinstone S.M., Alter H., Rice C.M., McKeating J.A. (2004). Neutralizing antibody response during acute and chronic hepatitis C virus infection. Proc. Natl. Acad. Sci. USA.

[19] Huang X.J., Lü X., Lei Y.F., Yang J., Yao M., Lan H.Y., Zhang J.M., Jia Z.S., Yin W., Xu Z.K. (2013). Cellular immunogenicity of a multi-epitope peptide vaccine candidate based on hepatitis C virus NS5A, NS4B and core proteins in HHD-2 mice. J. Virol. Methods.

[20] Martínez-Donato G., Piniella B., Aguilar D., Olivera S., Pérez A., Castañedo Y., Alvarez-Lajonchere L., Dueñas-Carrera S., Lee J.W., Burr N. (2016). Protective T Cell and Antibody Immune Responses against Hepatitis C Virus Achieved Using a Biopolyester-Bead-Based Vaccine Delivery System. Clin. Vaccine Immunol..

[21] Roohvand F., Aghasadeghi M.-R., Sadat S.M., Budkowska A., Khabiri A.-R. (2007). HCV core protein immunization with Montanide/CpG elicits strong Th1/Th2 and long-lived CTL responses. Biochem. Biophys. Res. Commun..

[22] Guest J.D., Pierce B.G. (2021). Structure-Based and Rational Design of a Hepatitis C Virus Vaccine. Viruses.

[23] Fuenmayor J., Godia F., Cervera L. (2017). Production of virus-like particles for vaccines. New Biotechnol..

[24] Gupta R., Arora K., Roy S.S., Joseph A., Rastogi R., Arora N.M., Kundu P.K. (2023). Platforms, advances, and technical challenges in virus-like particles-based vaccines. Front. Immunol..

[25] Baumert T.F., Ito S., Wong D.T., Liang T.J. (1998). Hepatitis C virus structural proteins assemble into viruslike particles in insect cells. J. Virol..

[26] Masavuli M.G., Wijesundara D.K., Torresi J., Gowans E.J., Grubor-Bauk B. (2017). Preclinical Development and Production of Virus-Like Particles as Vaccine Candidates for Hepatitis C. Front. Microbiol..

[27] Ghasemi F., Rostami S., Meshkat Z. (2015). Progress in the development of vaccines for hepatitis C virus infection. World J. Gastroenterol..

[28] Gomez-Escobar E., Roingeard P., Beaumont E. (2023). Current Hepatitis C Vaccine Candidates Based on the Induction of Neutralizing Antibodies. Viruses.

[29] Christiansen D., Earnest-Silveira L., Grubor-Bauk B., Wijesundara D.K., Boo I., Ramsland P.A., Vincan E., Drummer H.E., Gowans E.J., Torresi J. (2019). Pre-clinical evaluation of a quadrivalent HCV VLP vaccine in pigs following microneedle delivery. Sci. Rep..

[30] Lechmann M., Murata K., Satoi J., Vergalla J., Baumert T.F., Liang T.J. (2001). Hepatitis C virus-like particles induce virus-specific humoral and cellular immune responses in mice. Hepatology.

[31] Jeong S.H., Qiao M., Nascimbeni M., Hu Z., Rehermann B., Murthy K., Liang T.J. (2004). Immunization with hepatitis C virus-like particles induces humoral and cellular immune responses in nonhuman primates. J. Virol..

[32] Earnest-Silveira L., Christiansen D., Herrmann S., Ralph S.A., Das S., Gowans E.J., Torresi J. (2016). Large scale production of a mammalian cell derived quadrivalent hepatitis C virus like particle vaccine. J. Virol. Methods.

[33] Earnest-Silveira L., Chua B., Chin R., Christiansen D., Johnson D., Herrmann S., Ralph S.A., Vercauteren K., Mesalam A., Meuleman P. (2016). Characterization of a hepatitis C virus-like particle vaccine produced in a human hepatocyte-derived cell line. J. Gen. Virol..

[34] Polakos N.K., Drane D., Cox J., Ng P., Selby M.J., Chien D., O’Hagan D.T., Houghton M., Paliard X. (2001). Characterization of hepatitis C virus core-specific immune responses primed in rhesus macaques by a nonclassical ISCOM vaccine. J. Immunol..

[35] Drane D., Maraskovsky E., Gibson R., Mitchell S., Barnden M., Moskwa A., Shaw D., Gervase B., Coates S., Houghton M. (2009). Priming of CD4+ and CD8+ T cell responses using a HCV core ISCOMATRIX™ vaccine: A phase I study in healthy volunteers. Hum. Vaccines.

[36] Eshaghi B., Fofana J., Nodder S.B., Gummuluru S., Reinhard B.M. (2022). Virus-Mimicking Polymer Nanoparticles Targeting CD169+ Macrophages as Long-Acting Nanocarriers for Combination Antiretrovirals. ACS Appl. Mater. Interfaces.

[37] Liu H.-Y., Li X., Wang Z.-G., Liu S.-L. (2023). Virus-mimicking nanosystems: From design to biomedical applications. Chem. Soc. Rev..

[38] Somiya M., Kuroda S.I. (2015). Development of a virus-mimicking nanocarrier for drug delivery systems: The bio-nanocapsule. Adv. Drug Deliv. Rev..

[39] Li X., Liu S., Yin P., Chen K. (2022). Enhanced immune responses by virus-mimetic polymeric nanostructures against infectious diseases. Front. Immunol..

[40] Lou B., De Beuckelaer A., Boonstra E., Li D., De Geest B.G., De Koker S., Mastrobattista E., Hennink W.E. (2019). Modular core-shell polymeric nanoparticles mimicking viral structures for vaccination. J. Control. Release.

[41] Somiya M., Liu Q., Kuroda S.I. (2017). Current progress of virus-mimicking nanocarriers for drug delivery. Nanotheranostics.

[42] van Rijn P., Schirhagl R. (2016). Viruses, Artificial Viruses and Virus-Based Structures for Biomedical Applications. Adv. Healthcare Mater..

[43] Liu C., Xu H., Sun Y., Zhang X., Cheng H., Mao S. (2019). Design of Virus-Mimicking Polyelectrolyte Complexes for Enhanced Oral Insulin Delivery. J. Pharm. Sci..

[44] Lee C., Jeong J., Lee T., Zhang W., Xu L., Choi J.E., Park J.H., Song J.K., Jang S., Eom C.-Y. (2018). Virus-mimetic polymer nanoparticles displaying hemagglutinin as an adjuvant-free influenza vaccine. Biomaterials.

[45] DeCollibus D.P., Marin A., Andrianov A.K. (2010). Effect of Environmental Factors on Hydrolytic Degradation of Water-Soluble Polyphosphazene Polyelectrolyte in Aqueous Solutions. Biomacromolecules.

[46] Andrianov A.K., Langer R. (2021). Polyphosphazene immunoadjuvants: Historical perspective and recent advances. J. Control. Release.

[47] Chand D.J., Magiri R.B., Wilson H.L., Mutwiri G.K. (2021). Polyphosphazenes as Adjuvants for Animal Vaccines and Other Medical Applications. Front. Bioeng. Biotechnol..

[48] Hlushko R., Pozharski E., Prabhu V.M., Andrianov A.K. (2024). Directly visualizing individual polyorganophosphazenes and their single-chain complexes with proteins. Commun. Mater..

[49] Marin A., Kethanapalli S.H., Andrianov A.K. (2024). Immunopotentiating Polyphosphazene Delivery Systems: Supramolecular Self-Assembly and Stability in the Presence of Plasma Proteins. Mol. Pharm..

[50] Leyva-Grado V.H., Marin A., Hlushko R., Yunus A.S., Promeneur D., Luckay A., Lazaro G.G., Hamm S., Dimitrov A.S., Broder C.C. (2024). Nano-Assembled Polyphosphazene Delivery System Enables Effective Intranasal Immunization with Nipah Virus Subunit Vaccine. ACS Appl. Bio Mater..

[51] Andrianov A.K., Marin A., Fuerst T.R. (2016). Molecular-Level Interactions of Polyphosphazene Immunoadjuvants and Their Potential Role in Antigen Presentation and Cell Stimulation. Biomacromolecules.

[52] Vasilakos J.P., Tomai M.A. (2013). The use of Toll-like receptor 7/8 agonists as vaccine adjuvants. Expert Rev. Vaccines.

[53] Tomai M.A., Vasilakos J.P. (2011). TLR-7 and -8 agonists as vaccine adjuvants. Expert Rev. Vaccines.

[54] Bhagchandani S., Johnson J.A., Irvine D.J. (2021). Evolution of Toll-like receptor 7/8 agonist therapeutics and their delivery approaches: From antiviral formulations to vaccine adjuvants. Adv. Drug Deliv. Rev..

[55] Dowling D.J. (2018). Recent Advances in the Discovery and Delivery of TLR7/8 Agonists as Vaccine Adjuvants. ImmunoHorizons.

[56] Schindelin J., Arganda-Carreras I., Frise E., Kaynig V., Longair M., Pietzsch T., Preibisch S., Rueden C., Saalfeld S., Schmid B. (2012). Fiji: An open-source platform for biological-image analysis. Nat. Methods.

[57] Charan J., Kantharia N.D. (2013). How to calculate sample size in animal studies?. J. Pharmacol. Pharmacother..

[58] Midgard H., Bjøro B., Mæland A., Konopski Z., Kileng H., Damås J.K., Paulsen J., Heggelund L., Sandvei P.K., Ringstad J.O. (2016). Hepatitis C reinfection after sustained virological response. J. Hepatol..

[59] Andrianov A.K., Marin A., Wang R., Karauzum H., Chowdhury A., Agnihotri P., Yunus A.S., Mariuzza R.A., Fuerst T.R. (2020). Supramolecular assembly of Toll-like receptor 7/8 agonist into multimeric water-soluble constructs enables superior immune stimulation in vitro and in vivo. ACS Appl. Bio Mater..

[60] Messaud F.A., Sanderson R.D., Runyon J.R., Otte T., Pasch H., Williams S.K.R. (2009). An overview on field-flow fractionation techniques and their applications in the separation and characterization of polymers. Prog. Polym. Sci..

[61] Pitkänen L., Striegel A.M. (2014). AF4/MALS/QELS/DRI characterization of regular star polymers and their “span analogs”. Analyst.

[62] Andrianov A.K., Marin A., Wang R., Chowdhury A., Agnihotri P., Yunus A.S., Pierce B.G., Mariuzza R.A., Fuerst T.R. (2021). In Vivo and In Vitro Potency of Polyphosphazene Immunoadjuvants with Hepatitis C Virus Antigen and the Role of Their Supramolecular Assembly. Mol. Pharm..

[63] Weissenberger G., Henderikx R.J.M., Peters P.J. (2021). Understanding the invisible hands of sample preparation for cryo-EM. Nat. Methods.

[64] Yip K.M., Fischer N., Paknia E., Chari A., Stark H. (2020). Atomic-resolution protein structure determination by cryo-EM. Nature.

[65] Cheng Y. (2018). Single-particle cryo-EM-How did it get here and where will it go. Science.

[66] Gopal A., Zhou Z.H., Knobler C.M., Gelbart W.M. (2012). Visualizing large RNA molecules in solution. RNA.

[67] Lyu Z., Yao L., Chen W., Kalutantirige F.C., Chen Q. (2023). Electron Microscopy Studies of Soft Nanomaterials. Chem. Rev..

[68] Wang F., Gnewou O., Solemanifar A., Conticello V.P., Egelman E.H. (2022). Cryo-EM of Helical Polymers. Chem. Rev..

[69] Mai D.J., Schroeder C.M. (2020). 100th Anniversary of Macromolecular Science Viewpoint: Single-Molecule Studies of Synthetic Polymers. ACS Macro Lett..

[70] Parry A.L., Bomans P.H.H., Holder S.J., Sommerdijk N.A.J.M., Biagini S.C.G. (2008). Cryo Electron Tomography Reveals Confined Complex Morphologies of Tripeptide-Containing Amphiphilic Double-Comb Diblock Copolymers. Angew. Chem. Int. Ed..

[71] Wu H., Ting J.M., Tirrell M.V. (2020). Mechanism of Dissociation Kinetics in Polyelectrolyte Complex Micelles. Macromolecules.

[72] Lueckheide M., Vieregg J.R., Bologna A.J., Leon L., Tirrell M.V. (2018). Structure–Property Relationships of Oligonucleotide Polyelectrolyte Complex Micelles. Nano Lett..

[73] Marras A.E., Vieregg J.R., Ting J.M., Rubien J.D., Tirrell M.V. (2019). Polyelectrolyte Complexation of Oligonucleotides by Charged Hydrophobic—Neutral Hydrophilic Block Copolymers. Polymers.

[74] Yu X., Qiao M., Atanasov I., Hu Z., Kato T., Liang T.J., Zhou Z.H. (2007). Cryo-electron microscopy and three-dimensional reconstructions of hepatitis C virus particles. Virology.

[75] Seder R.A., Darrah P.A., Roederer M. (2008). T-cell quality in memory and protection: Implications for vaccine design. Nat. Rev. Immunol..

[76] Andrianov A.K., Fuerst T.R. (2021). Immunopotentiating and Delivery Systems for HCV Vaccines. Viruses.

[77] Landi A., Law J., Hockman D., Logan M., Crawford K., Chen C., Kundu J., Ebensen T., Guzman C.A., Deschatelets L. (2017). Superior immunogenicity of HCV envelope glycoproteins when adjuvanted with cyclic-di-AMP, a STING activator or archaeosomes. Vaccine.

[78] Bouveret Le Cam N.N., Ronco J., Francon A., Blondeau C., Fanget B. (1998). Adjuvants for influenza vaccine. Res. Immunol..

[79] Ison M.G., Mills J., Openshaw P., Zambon M., Osterhaus A., Hayden F. (2002). Current research on respiratory viral infections: Fourth International Symposium. Antivir. Res..

[80] O’Connell R.J., Excler J.-L., Polonis V.R., Ratto-Kim S., Cox J., Jagodzinski L.L., Liu M., Wieczorek L., McNeil J.G., El-Habib R. (2016). Safety and Immunogenicity of a randomized Phase I prime-boost trial with ALVAC-HIV (vCP205) and Oligomeric gp160 MN/LAI-2 Adjuvanted in Alum or Polyphosphazene. J. Infect. Dis..

[81] O’Connell R., Polonis V., Ratto-Kim S., Cox J., Jagodzinski L., Malia J., Michael N., Excler J., Robb M., Kim J. (2012). Safety and immunogenicity of a randomized phase I prime-boost trial with ALVAC-HIV (vCP205) and gp160 MN/LAI-2 adjuvanted in alum or polyphosphazene. Retrovirology.

[82] Laera D., HogenEsch H., O’Hagan D.T. (2023). Aluminum Adjuvants—‘Back to the Future’. Pharmaceutics.

[83] Zhang T., He P., Guo D., Chen K., Hu Z., Zou Y. (2023). Research Progress of Aluminum Phosphate Adjuvants and Their Action Mechanisms. Pharmaceutics.

[84] Jully V., Mathot F., Moniotte N., Préat V., Lemoine D. (2016). Mechanisms of Antigen Adsorption onto an Aluminum-Hydroxide Adjuvant Evaluated by High-Throughput Screening. J. Pharm. Sci..

[85] Lu Y., Liu G. (2022). Nano alum: A new solution to the new challenge. Hum. Vaccines Immunother..

[86] Kurzątkowski W., Kartoğlu Ü., Górska P., Główka M., Woźnica K., Zasada A.A., Szczepańska G., Trykowski G., Gniadek M., Donten M. (2018). Physical and chemical changes in Alhydrogel™ damaged by freezing. Vaccine.

[87] Maughan C.N., Preston S.G., Williams G.R. (2015). Particulate inorganic adjuvants: Recent developments and future outlook. J. Pharm. Pharmacol..

[88] Pierce B.G., Keck Z.-Y., Wang R., Lau P., Garagusi K., Elkholy K., Toth E.A., Urbanowicz R.A., Guest J.D., Agnihotri P. (2020). Structure-based design of hepatitis C virus E2 glycoprotein improves serum binding and cross-neutralization. J. Virol..

[89] Reed S.G., Orr M.T., Fox C.B. (2013). Key roles of adjuvants in modern vaccines. Nat. Med..

[90] Kayesh M.E.H., Kohara M., Tsukiyama-Kohara K. (2023). TLR agonists as vaccine adjuvants in the prevention of viral infections: An overview. Front. Microbiol..

[91] Kannanganat S., Ibegbu C., Chennareddi L., Robinson H.L., Amara R.R. (2007). Multiple-cytokine-producing antiviral CD4 T cells are functionally superior to single-cytokine-producing cells. J Virol.

[92] Pierce B.G., Felbinger N., Metcalf M., Toth E.A., Ofek G., Fuerst T.R. (2024). Hepatitis C Virus E1E2 Structure, Diversity, and Implications for Vaccine Development. Viruses.

[93] Vijayamahantesh V., Patra T., Meyer K., Alameh M.G., Reagan E.K., Weissman D., Ray R. (2022). Modified E2 Glycoprotein of Hepatitis C Virus Enhances Proinflammatory Cytokines and Protective Immune Response. J. Virol..

[94] Andrianov A.K., Chen J., LeGolvan M.P. (2004). Poly(dichlorophosphazene) as a precursor for biologically active polyphosphazenes: Synthesis, characterization, and stabilization. Macromolecules.

