# Supplementary material for: Virus-Mimicking Polymer Nanocomplexes Co-Assembling HCV E1E2 and Core Proteins with TLR 7/8 Agonist—Synthesis, Characterization, and In Vivo Activity
Source: J Funct Biomater. 2025 Jan 19;16(1):34. doi: 10.3390/jfb16010034 (PMC11766188; doi:10.3390/jfb16010034)
Supplement: Supplementary file 1 [file jfb-16-00034-s001.zip › jfb-3410753-supplementary.pdf]

Virus-Mimicking Polymer Nanocomplexes Co-Assembling HCV E1E2 and Core Proteins with TLR 7/8 Agonist – Synthesis, Characterization and *In Vivo* Activity

Thomas R. Fuerst [1,2,†,*], Alexander Marin [1], Sarah Jeong [1], Liudmila Kulakova [1], Raman Hlushko [1], Katrina Gorga [3], Eric A. Toth [1], Nevil J. Singh [3] and Alexander K. Andrianov [1,†,*]

[1] Institute for Bioscience and Biotechnology Research, University of Maryland Rockville, MD 20850, USA
[2] Department of Cell Biology and Molecular Genetics, University of Maryland, College Park, MD 20742, USA
[3] Department of Microbiology & Immunology, University of Maryland School of Medicine, Baltimore, MD 21201, USA

† These authors contributed equally
* Correspondence: tfuerst@umd.edu (T.R.F.), aandrianov@umd.edu (A.K.A.)

Supplementary Materials

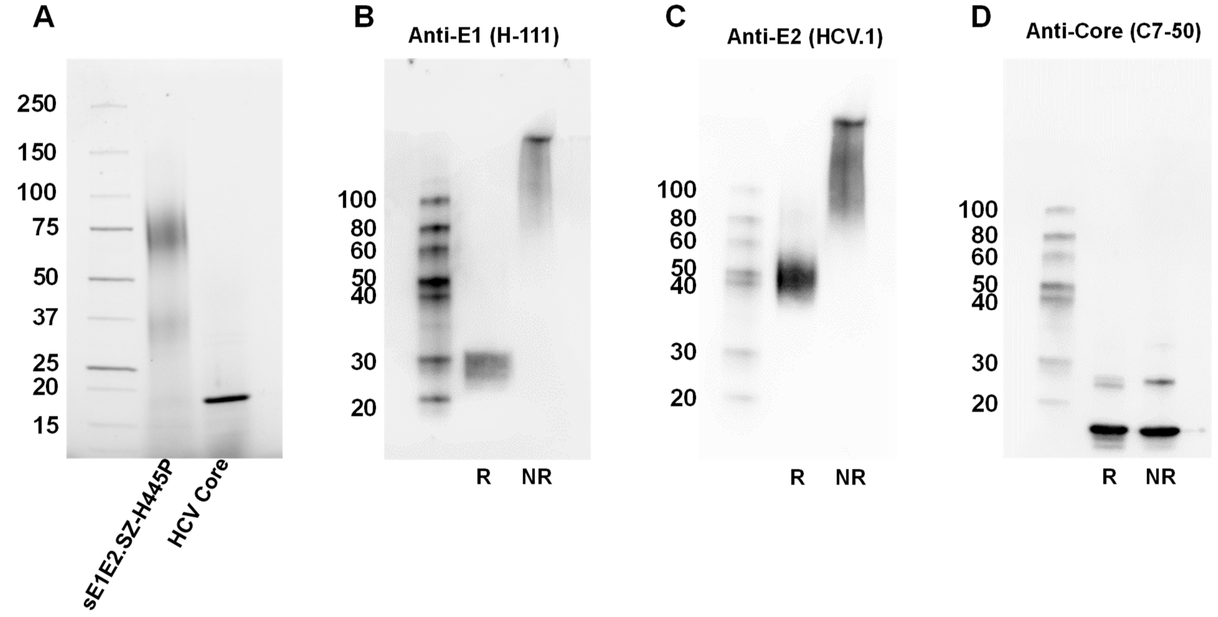

Figure S1. Characterization of sE1E2.SZ-H445P and HCV core antigens. (A) SDS/PAGE analysis of purified sE1E2SZ.H445P and HCV.1 Core under reducing conditions, (B) Western blot detection of purified sE1E2.SZ-H445P under reducing (lane R) and non-reducing (lane NR) conditions using anti-E1 mAb (H-111), (C) Western blot detection of purified sE1E2SZ.H445P under reducing (R) and non-reducing (NR) conditions using anti-E2 mAb (HCV.1), and (D) Western blot detection of purified HCV Core under reducing (R) and non-reducing (NR) conditions using anti-Core mAb (C7-50).

Synthesis of PCPP-PEG

Materials: Methoxypolyethylene glycol amine (5000 g/mol), PEG-NH_2, anhydrous diglyme, triethylamine, sodium hydride, potassium hydroxide, (MilliporeSigma, St. Louis, MO), ethanol (The Warner-Graham Company, Cockeysville, MD) and propylparaben (Spectrum Chemical, Gardena, CA) were used as received. Polydichlorophosphazene, PDCP was synthesized as described previously [1].

Synthesis: All procedures involving PDCP were carried out under an atmosphere of dry nitrogen using MBraun Labstar Pro glovebox workstation (M. Braun Inertgas-Systeme GMBH, Garching, Germany) or common air-free laboratory techniques.

PEG-NH_2 (1.25 g, 0.25 mmol) was dissolved in an anhydrous diglyme (80 mL) at 50 °C under stirring and triethylamine (35 μL, 0.25 mmol) was added to this solution. The resulting mixture was added dropwise to a solution of PDCP (0.464 g, 4.0 mmol) in diglyme (70 mL) at 50 °C, the reaction was brought to ambient temperature and stirred overnight.

Propylparaben (2.9 g, 16.0 mmol) was dissolved in diglyme (40 mL) and heated under nitrogen at 120 °C for 30 minutes. The heat was removed, and once the reaction mixture had cooled, a suspension of sodium hydride (0.365 g, 15.2 mmol) in an anhydrous diglyme (15 mL) was added dropwise. The solution was allowed to stir at ambient temperature for 1 h.

The solution of sodium propylparaben was then added to the PDCP-PEG-NH_2 reaction mixture at 50 °C under stirring. The temperature of the reaction was increased to 120 °C and stirring continued for 3 h. Heating was removed, and potassium hydroxide (20 mL) was added once the temperature fell below 100 °C. The mixture was stored at 4 °C overnight. The polymer was collected by centrifugation, dissolved in deionized water, and precipitated with ethanol. It was then redissolved in deionized water, dialyzed using a 50 kDa cutoff cellulose membrane, and lyophilized. The yield was 1.1 g (59%).

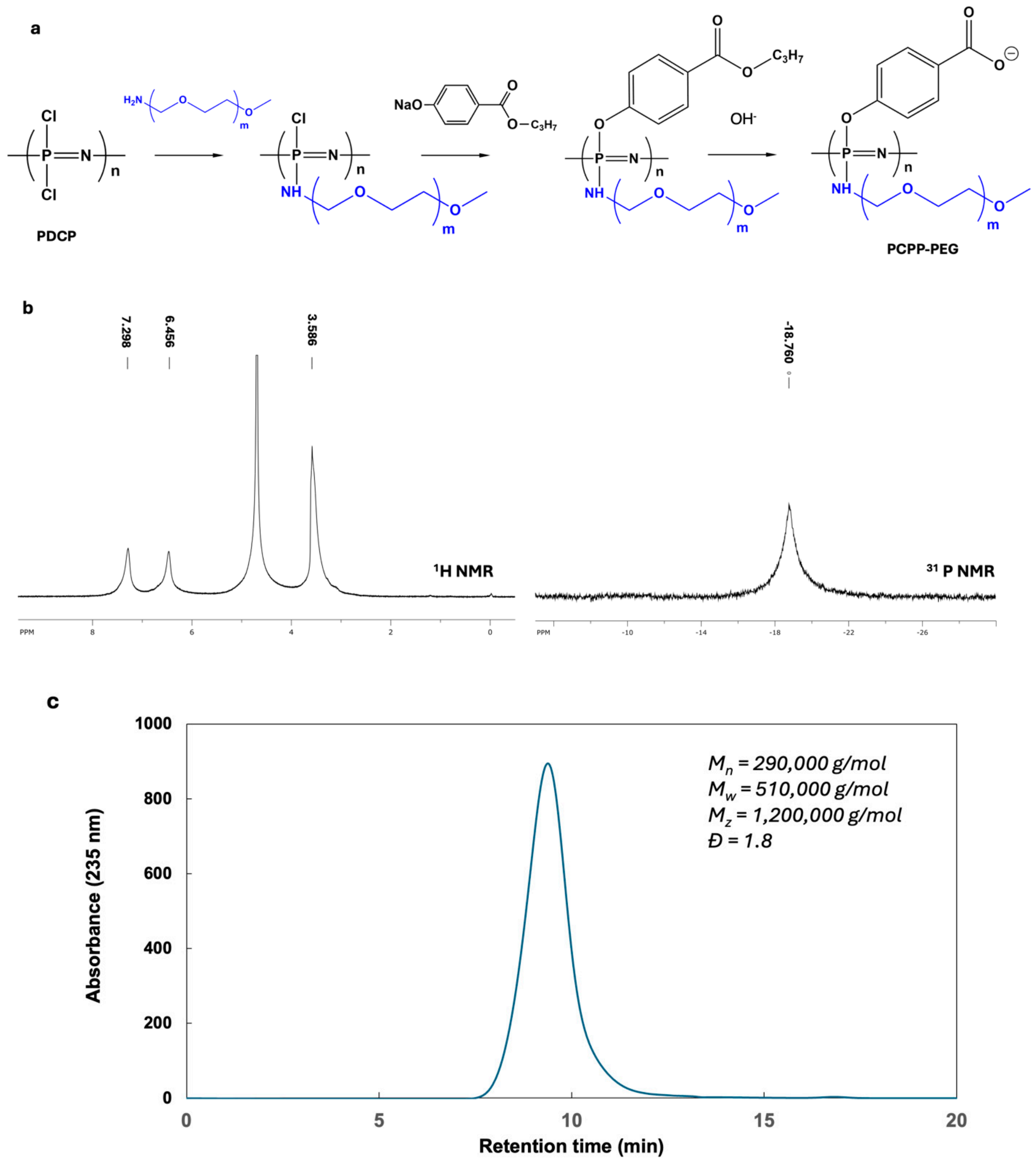

Figure S2. Synthesis and physico-chemical characterization of PCPP-PEG. (a) Synthetic pathway to PCPP-PEG, (b) ^{1}H NMR (400 MHz, D_2O, pH 7.4) and ^{31}P NMR (161.9 MHz, pH 7.4) spectra of PCPP-PEG and (c) size exclusion chromatogram of PCPP-PEG (phosphate buffer, pH 7.4, UV detection).

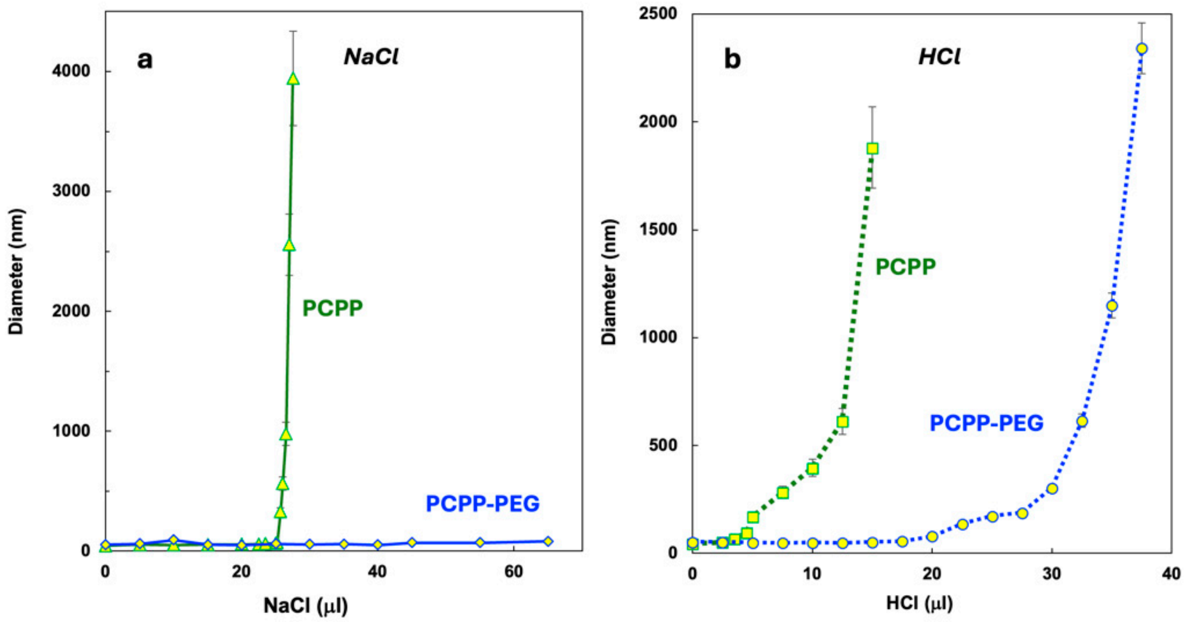

Figure S3. DLS studies demonstrating superior environmental stability of PCPP-PEG over PCPP. Hydrodynamic diameter of PCPP and PCPP-PEG as a result of titration with (a) sodium chloride and (b) hydrochloric acid (200 L of 2 mg/mL polymer in PBS; titration with 20% (w/w) sodium chloride and 0.05 M hydrochloric acid)

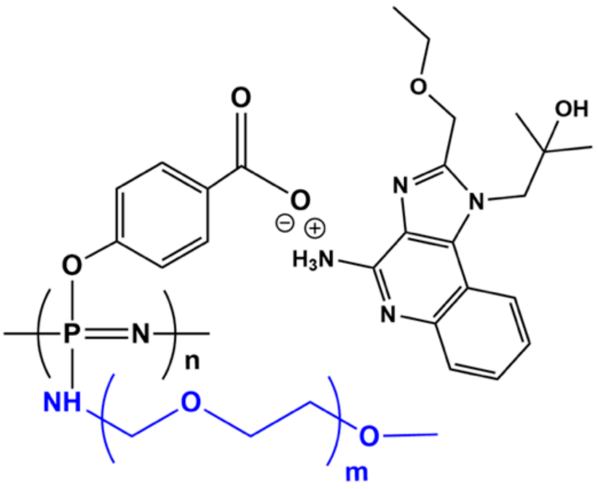

Figure S4. Ionic binding of R848 to a PCPP-PEG side chain.

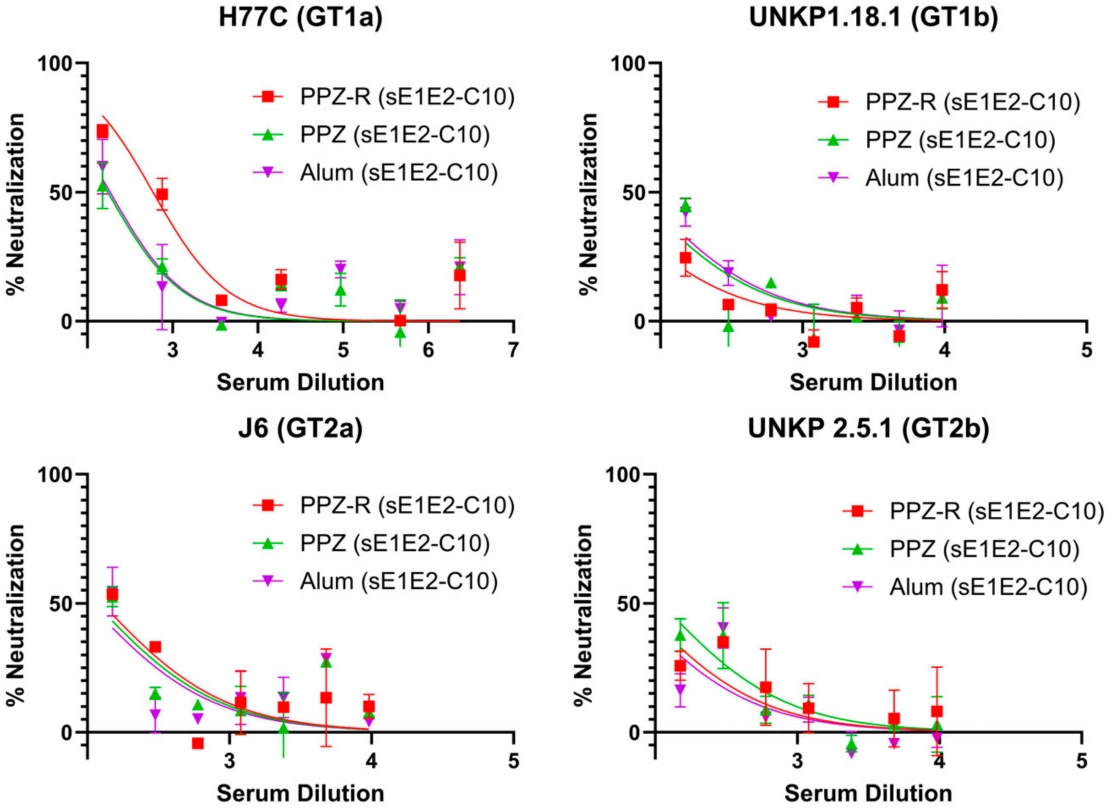

Figure S5. Breadth of neutralization against 4 core genotypes with HCVpp. (A-D) Pooled immunized mice sera were assessed for neutralization activities at day 56 and day 0 against 4 genotypes of HCVpp. Percent neutralization was calculated based on the reduction of serum RLU (Relative Light Units) relative to the Day 0 neutralization, using curve fitting in GraphPad Prism software. Serum dilutions were performed as five-fold dilutions for H77C (GT1a) and two-fold dilutions for UNKP1.18.1 (GT1b), J6 (GT2a), and UNKP2.5.1 (GT2b) starting at 1:150 for HCVpp neutralization. The experiment was performed in duplicate and the error bars represent the standard deviation (SD).

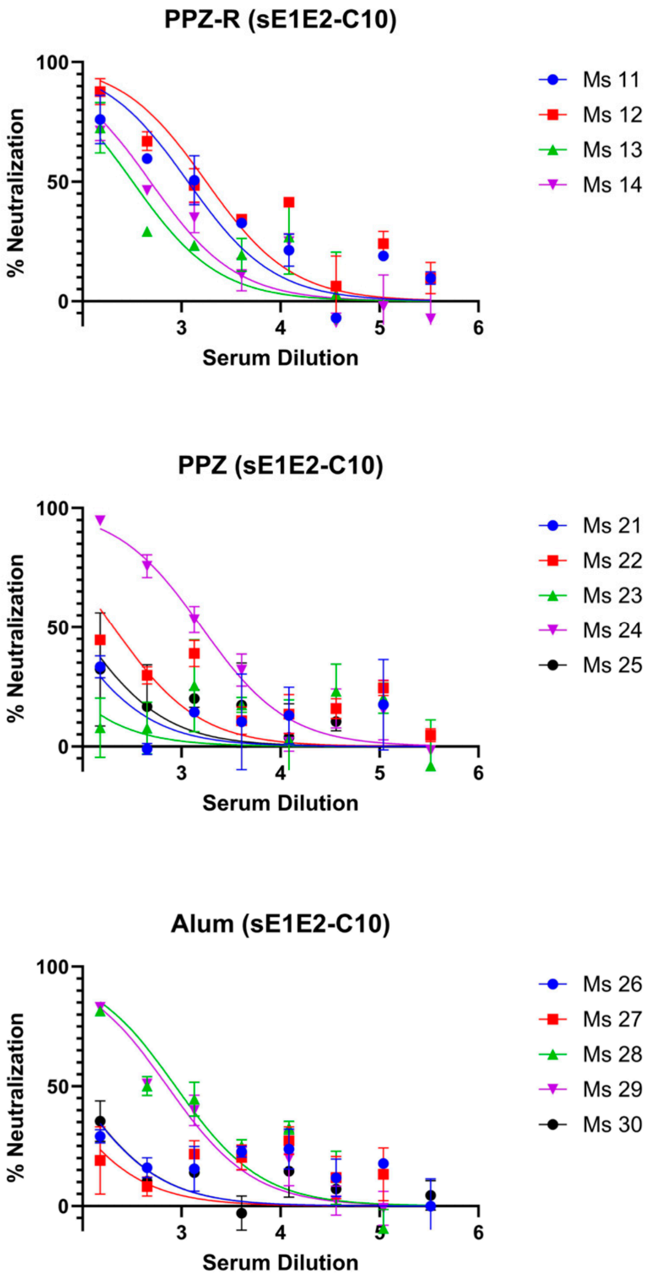

Figure S6. Breadth of neutralization of individual mouse sera against H77C (GT1a). (A-D) Individual immunized mice sera were assessed for neutralization activities at day 56 and day 0. Percent neutralization was calculated based on the reduction of serum RLU (Relative Light Units) relative to the RLU of the 100% control containing PBS and no serum, using curve fitting in GraphPad Prism software. Serum dilutions were performed as three-fold dilutions starting at 1:150 for HCVpp neutralization. The experiment was performed in duplicate and the error bars represent the standard deviation (SD).

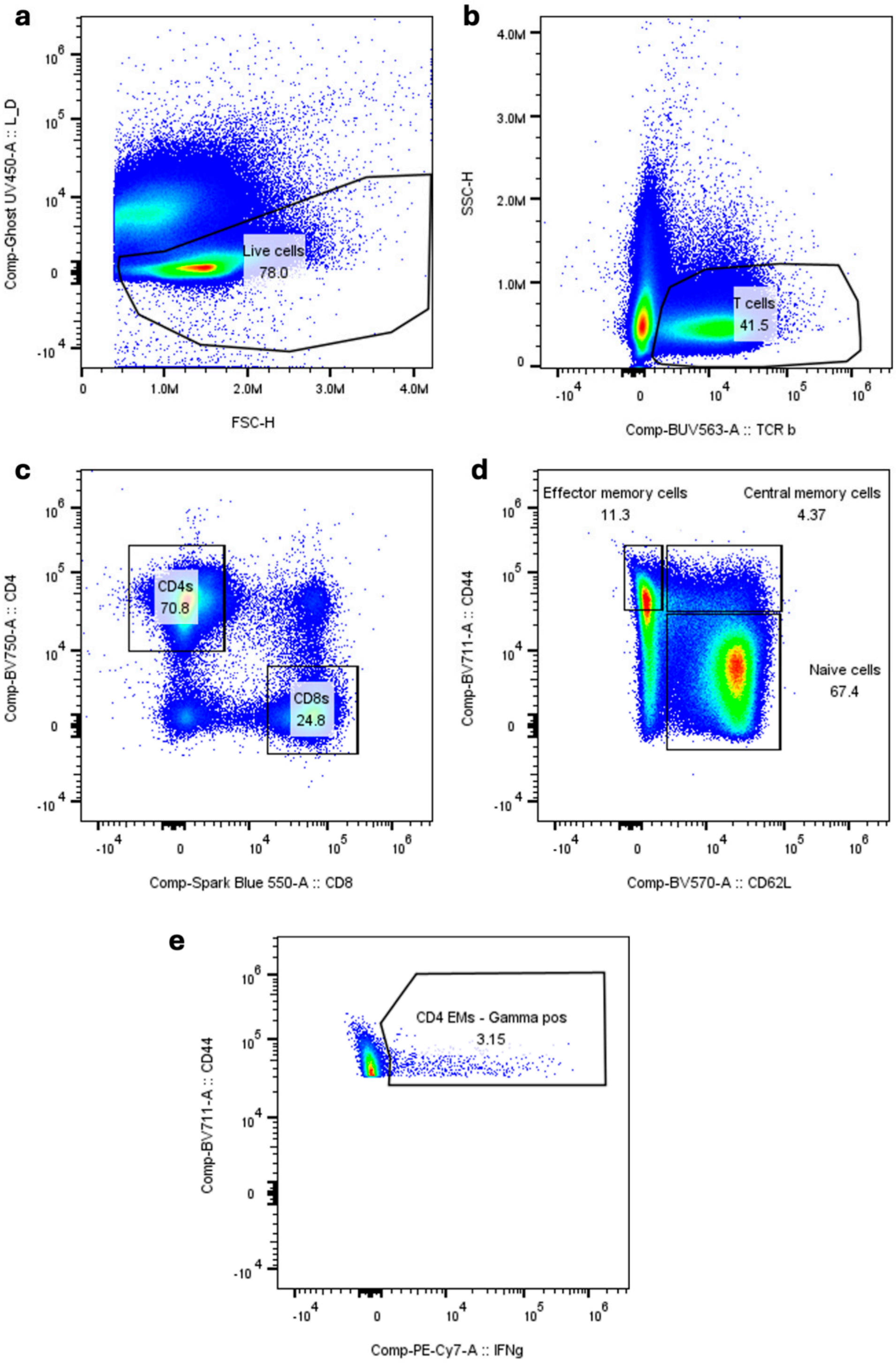

Figure S7. Gating strategy. Live cells, gated by exclusion of a Ghost-UV450 live-dead dye (a) were then gated on TCR-β positive cells (b) to identify T cells. This population was further sectioned into CD4 and CD8 positive cells (c). Within each subset, the CD44 and CD62L expression was used to identify effector/memory phenotype cells (CD44-hi, CD27L-low (d) before examining IFN-γ production in the cohort (e).

Reference

1. Andrianov, A. K.; Chen, J.; LeGolvan, M. P., Poly(dichlorophosphazene) as a precursor for biologically active polyphosphazenes: Synthesis, characterization, and stabilization. *Macromolecules* **2004,** 37, (2), 414-420.
